# Proteomics Analysis of Changes in the Water-Holding Capacity of Yak Meat During Postmortem Aging

**DOI:** 10.3390/foods15101652

**Published:** 2026-05-09

**Authors:** Zhaobin Guo, Xixiong Shi, Yubin Zhang, Cheng Chen, Guoyuan Ma, Long He, Li Zhang

**Affiliations:** College of Food Science and Engineering, Gansu Agricultural University, Lanzhou 730070, China

**Keywords:** yak meat, water-holding capacity, proteomics, key proteins, bioinformatics analysis

## Abstract

To elucidate the mechanism underlying changes in the water-holding capacity of yak meat during postmortem aging, yak longissimus dorsi muscle was used as the experimental material. Changes in pressure loss, drip loss, cooking loss, and pH were determined at 0, 0.5, 1, 3, 5, and 7 d postmortem (0–4 °C; RH 80–85%). Proteomic approaches were employed to identify key proteins associated with water-holding capacity and the related metabolic pathways at representative aging stages. The results showed that the pressure loss, drip loss, and cooking loss of yak meat all exhibited an initial increase followed by a decrease during the 7 d postmortem aging period, reaching their maximum values at 3 d (42.10%, 3.19%, and 42.82%. *p* < 0.05). In contrast, pH displayed an opposite trend and declined to the minimum value of 5.35 at 3 d. Furthermore, proteomics analysis conducted at 0, 3 and 5 d of aging identified a total of 1239 proteins and screened 41 differentially expressed proteins. Through correlation analysis, 14 key proteins significantly associated with the WHC of yak meat were identified. Protein–protein interaction analysis indicated that Titin isoform X3, Calpastatin isoform I, and small muscular protein might serve as indicator proteins for the WHC of yak meat during aging. Most of these proteins are metabolic enzymes, structural proteins and stress proteins, which may influence meat quality by affecting the microstructure and metabolic pathways of the muscle.

## 1. Introduction

The yak (*Bos grunniens*) is a unique mammalian species primarily distributed in high-altitude regions above 3000 m, such as the Himalayas and the Qinghai–Tibet Plateau [1]. In China, yaks are mainly found in alpine meadow regions with low pressure, long- cold seasons, and short warm seasons, such as southern Xinjiang, Qinghai, northwestern Gansu, and Xizang, accounting for over 90% of the world’s total yak population. Their species and origin differ from those of any yellow cattle or beef cattle, making them one of the three original species in the world [2]. Yak meat is characterized by high protein, low fat, a relatively complete range of essential and non-essential amino acids, and high mineral content (especially iron), making it a naturally green food product widely favored by consumers [3]. In recent years, yak meat production enterprises in the Qinghai–Tibet Plateau region have developed specialty products such as yak cut meat, chilled meat, hotpot slices and air-dried yak meat. These products are sold nationwide. Additionally, with the increased international exchanges and cooperation, the proportion of yak meat in the global meat consumption market is also on the rise [4].

The water-holding capacity (WHC) of meat is one of the most crucial indicators for consumers to evaluate the quality of meat. It not only directly affects the juiciness, tenderness, color, and flavor of the meat, but also has significant economic implications [5]. The WHC of chilled fresh meat produced in China is relatively low, which affects the beef quality and shelf life. According to statistics, the juice loss rate of chilled fresh meat ranges from 1% to 3%, resulting in direct economic losses for enterprises exceeding RMB 5 billion annually [6].

Commonly used methods for determining the WHC of meat include the pressure loss method, near-infrared spectroscopy, low-field nuclear magnetic resonance (LF-NMR), and conductivity method [7]. These methods can only qualitatively reflect the WHC of meat or characterize the existence state and migration process of different moisture groups in the meat, unable to reveal the molecular-level regulatory mechanism. In recent years, although proteomics technology has made significant progress in the field of meat science, there have been few reports on the use of proteomics to research the mechanisms of WHC changes during the postmortem aging of yak meat [8]. Yaks have lived in alpine hypoxic environments for a long time, and there are significant differences in muscle physiology between yaks and ordinary cattle. Therefore, the water retention mechanism of ordinary cattle cannot be directly applied. Targeted research must be conducted to form a quality control theory and technology suitable for yaks. The integrated scheme of TMT-labeled quantitative proteomics + bioinformatics + PRM verification was used to realize the whole chain analysis from protein identification, differential screening, key protein interaction to pathway enrichment, which was more accurate and higher flux than traditional 2D-DIGE, and filled the blank of high-depth quantitative proteomics research of yak meat. Luca et al. investigated the differentially expressed proteins in the centrifugal exudate of meat samples with varying drip loss. The results showed that 16 differentially expressed proteins were identified in the centrifugal exudate of the two groups of meat samples; in the group with higher drip loss, the expression levels of differential proteins were lower, playing an important role in maintaining cell integrity [9]. Zuo et al. analyzed the changes in WHC across different cooking loss groups of yak meat. The results showed that 10 proteins, including malate dehydrogenase, myosin heavy chain 1, and actin, had a significant impact on the WHC of yak meat. The main biological processes involved included protein degradation and denaturation, the tricarboxylic acid cycle, and anaerobic glycolysis [10]. Ertbjerg et al. demonstrated that desmin and creatine phosphokinase were potential biomarkers for pork with high drip loss; overexpression of creatine phosphokinase in high drip loss samples leads to rapid muscle contraction, thereby increasing drip loss [11]. Currently, there is no systematic explanation of the postmortem WHC mechanism in yak meat; protein is an important component of muscle, and changes in various muscle cell proteins, such as protein degradation, oxidation, and denaturation, can cause changes in the internal structure of muscle cells, thereby affecting the muscle WHC [12]. Proteins such as metabolic enzymes, structural proteins and stress proteins may change the water-holding capacity of meat by affecting the microstructure and metabolic pathways of muscle [13].

This study used yak longissimus dorsi muscle as the experimental material. Based on the dynamic changes in conventional WHC indices of yak meat during 0–7 d of postmortem aging, we selected three critical time points (0, 3, and 5 d) with significant differences in WHC. Tandem mass tag (TMT)-based quantitative proteomics integrated with bioinformatic analysis was applied to explore the molecular mechanism underlying WHC changes during postmortem aging. The key proteins responsible for WHC alterations at different aging stages were screened, and the major metabolic pathways in which these key proteins participate were identified. This study aims to provide a scientific basis and theoretical guidance for refining the mechanism of meat WHC and supporting the deep processing and quality improvement of yak meat products.

## 2. Materials and Methods

### 2.1. Meat Samples and Processing

Ten male yaks aged 36–48 months, fed on the same diet and in the same batch, were obtained from Qinghai Baide Investment Development Co. Ltd. (Xining, China). The animals were fasted and deprived of water for 24 h before slaughter. After slaughtering, the carcasses were immediately subjected to decontamination treatment, and the longissimus dorsi muscle on the same side of the carcass was immediately removed, vacuum-packed, and transferred to the laboratory at 4 °C. After removing visible connective tissues, tendons and surface fat, the muscles were cut into approximately 100 g samples and stored at 4 °C for 0, 0.5, 1, 3, 5, and 7 days. Portions of samples randomly taken from each storage period were used to determine the conventional WHC and pH values. Additionally, the remainder of samples for 0, 3, and 5 days were immediately frozen in liquid nitrogen and stored at −80 °C in the laboratory for TMT-based quantitative proteomics analysis. Ethical approval was obtained prior to the experiment. All animal procedures were performed in accordance with the Guidelines for the Care and Use of Laboratory Animals of Gansu Agricultural University (GSAU-Eth-FSE-2024-006).

### 2.2. Determination of Conventional WHC

#### 2.2.1. Pressure Loss (PL)

Following the pressure filter paper method described by Li et al. [14]. A meat sample with a thickness of 1 cm was taken, and a 5.0 cm^2^ subsample was excised from the center using a stainless-steel cutter on a stainless-steel operating surface; this was then weighed and recorded as *M*1. The subsample was sandwiched between two layers of medical gauze, with 15–18 layers of absorbent filter paper placed above and below the meat sample. The assembly was placed on a compression platform and subjected to a uniform force of 35 kg for 5 min. After removal of the pressure, the meat sample was immediately weighed and recorded as *M*2. The formula for calculating the PL is as follows:

PL(%)=(M1−M2)/M1×100

#### 2.2.2. Drip Loss (DL)

The DL was measured as described by Bowker et al. [15]. Approximately 20 g of the LD muscle was excised along the direction of the muscle fibers and weighed and recorded as *W*1. The sample was hung in a polyvinyl chloride (PVC) bag and sealed tightly to prevent the meat sample from coming into contact with the packaging. The bag was hung in an environment at 0–4 °C. After aging for the specified time points, the sample was removed, its surface moisture was blotted dry with filter paper, and it was weighed and recorded as *W*2. The formula for calculating the DL is as follows:

DL(%)=(W1−W2)/W1×100

#### 2.2.3. Cooking Loss (CL)

The CL was measured as described by Zhang et al. [16]. Raw meat samples (50 g) were weighed and recorded as *X*1; then, they were placed in retort pouch and cooked at a constant temperature in a water bathwater bath at 90 °C. When the center temperature of the meat sample reached 70 °C, held for 20 min, the sample was cooled to room temperature (20 °C) and weighed and recorded as *X*2. The formula for calculating the CL is as follows:

CL(%)=(X1−X2)/X1×100

#### 2.2.4. pH Value

pH values were assessed by a pH meter (HI 99,163 model) with the electrode inserted into raw meat samples on corresponding aging time points and storage at 4 °C.

### 2.3. Protein Extraction and Peptide Enzymatic Digestion

The assay was performed as described by Yang et al. with slight modifications [17]. The samples were first ground into powder using liquid nitrogen; then, the proteins were extracted using SDT buffer (4% SDS, 100 mM Tris-HCl, 100 mM DTT, pH 7.2). Protein concentrations were quantified using the bicinchoninic acid (BCA) method. Approximately 200 μg of each protein sample was subjected to trypsin digestion using the filter-aided proteome preparation (FASP) method. The digested peptide segments were desalted using a C18 cartridge. A measure of 40 μL of the dissolution buffer was used to re-suspend 2 μg of the peptide segments; the mixture was shaken at 800 rpm for 2 min and centrifuged at 10,000× *g* for 15 min. The filtrate was collected for peptide quantification (OD280). Peptide segments weighing 200 μg from each sample were labeled according to the instructions of the TMT labeling kit, as shown in Table 1.

### 2.4. SCX Chromatographic Grading

The peptide segments were labeled with TMT reagent and then mixed together. They were fractionated using AKTA Purifier 100 system. Buffer A (0.01 M KH_2_PO_4_, 25% ACN, pH 3.0); Buffer B (0.01 M KH_2_PO_4_, 0.5 M KCl, 25% ACN, pH 3.0). Buffer A was the equilibration solution for the chromatographic column and the flow rate was 800 nL/min. The liquid chromatographic gradient was as follows: 0–22 min for the B solution linear gradient from 0 to 25%; 22–40 min for the linear gradient from 25 to 30%; 40–42 min for the linear gradient from 30 to 55%; 42–55 min for the linear gradient from 55 to 100%; 55–60 min for the B solution maintained at 100%; after 60 min, the B solution was reset to 0%.

### 2.5. Analysis of Enzymatic Hydrolysis Products by LC-MS/MS

Following chromatographic separation, the samples were subjected to mass spectrometry analysis. The analysis duration was 60 min, with detection performed in positive ion mode; the scan range of the parent ion was 300–1800 m/z, the resolution of the primary mass spectrum was 70,000 at 200 m/z, the maximum injection time was 10 ms, and the dynamic exclusion time was set to 40 s. The method for collecting the mass-to-charge ratio of peptides and peptide fragments was as follows: we collected 10 fragmentation spectra; the MS2 activation type was higher-energy collisional dissociation (HCD); the isolation window was 2 m/z; the resolution of the secondary mass spectrum was 17,500 at 200 m/z; the normalized collision energy was 30 eV; the underfill ratio was 0.1%.

### 2.6. Protein Identification and Quantitative Analysis

The raw data was subjected to mass spectrometry analysis and identified using Proteome Discoverer 1.4 software. The relevant analysis parameters were shown in Table 2.

### 2.7. Bioinformatics Analysis

#### 2.7.1. GO Annotation and Function Analysis

The Omicsbean version 2.0 software was used to conduct GO analysis on the target protein set. Each protein was annotated according to biological process (BP), cellular component (CC), and molecular function (MF). Based on the GO annotation results, the differentially expressed proteins between different treatment groups and the protein function entries significantly enriched by the target proteins were determined.

#### 2.7.2. KEGG Annotation and Enrichment Analysis

Using the Omicsbean version 2.0 software and the entire KEGG database, the KEGG annotation analysis was conducted on the target protein. Based on the KEGG annotation results, the significantly enriched pathways and metabolic pathways for the differentially expressed proteins and the target protein were determined.

#### 2.7.3. Protein Interaction Analysis

The STRING database (http://string-db.org) was used to identify protein–protein interaction (PPI) relationships among the target proteins, and a protein–protein interaction network diagram of the target proteins was generated and analyzed.

### 2.8. Statistical Analysis

Each assay was performed at least thrice, and the results were expressed as mean ± standard deviation (M ± SD). Statistical analyses were performed using SPSS version 24.0 (IBM, Armonk, New York, NY, USA). Differences among samples were evaluated by one-way analysis of variance (ANOVA) followed by Duncan’s multiple range test, with statistical significance defined at *p* < 0.05. Data processing and graphical visualization were carried out using Origin version 9.0 software. Pearson’s correlation analysis was conducted between WHC index and differential protein expression levels of the yak LD muscle to identify the key proteins significantly correlated with WHC. The software Mascot version 2.2 and Proteome Discoverer version 1.4 were used for database search and quantitative analysis in mass spectrometry analysis. Hierarchical clustering analysis of the identified differentially expressed proteins was performed using Cluster version 3.0 software. GO and KEGG enrichment analyses of key proteins were carried out using Omicsbean version 2.0 software. Protein–protein interaction network analysis was conducted based on information retrieved from the STRING (http://string-db.org/) database. The PRM validation was performed using the Skyline version 3.5.0 software to analyze the data of the PRM original file.

## 3. Results and Discussion

### 3.1. Changes in WHC

#### 3.1.1. Changes in Pressure Loss

**Figure 1:**
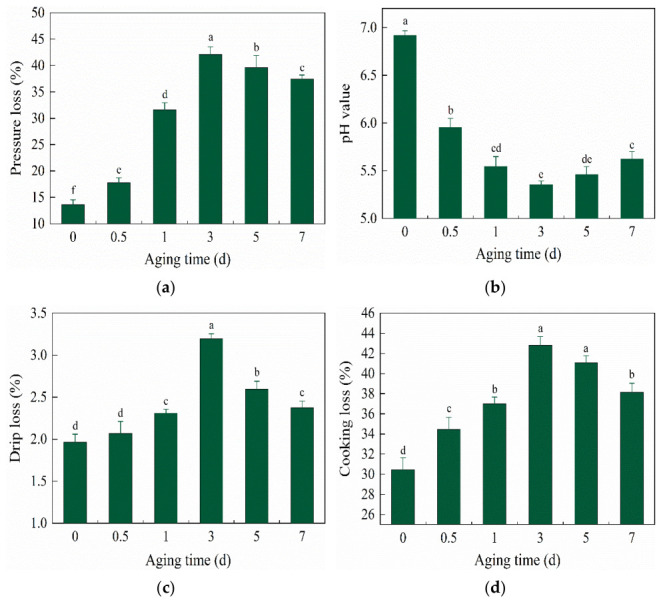
Change in WHC and pH value in yak meat during aging: (**a**) pressure loss; (**b**) pH value; (**c**) drip loss; (**d**) cooking loss. The lowercase letters represent the difference within the yak (*p* < 0.05).

The changes in PL during the postmortem aging period were shown in Figure 1a. As time increased, PL initially increased and then decreased. From 0 to 3 d, the PL increased significantly (*p* < 0.05), reaching a maximum value of 42.10% at 3 d, which was 28.48% higher than that at 0 d. After 3 d, it gradually decreased and dropped to 37.46% at 7 d. A greater PL indicates poorer WHC of the muscle [18]. Most of the WHC proteins within the muscle are located inside the cells, and these proteins possess a certain capacity to bind water. During the postmortem aging period, the water inside the cells is not easily lost. However, under the pressure force, this water will flow out of the cells, which can reflect the WHC of the muscle cells [19]. Under pressurized conditions, hydrogen bonds generally remain relatively stable, whereas hydrophobic interactions are disrupted, and covalent bonds are also affected to some extent [20]. In meat tissue, water is contained both within and between muscle fibers. Therefore, the water molecules inside and outside the protein matrix play a critical role in the interaction between proteins and water, directly influencing the WHC of the meat sample [6].

As shown in Figure 1b, the pH value reached its maximum of 6.92 at 0 d postmortem and decreased to a minimum of 5.35 at 3 d, which is referred to as the limit pH value, with a decrease of 22.69%. At this time, the PL was also at its maximum, because the muscle of the yak began to stiffen after slaughter, and glycogen underwent anaerobic glycolysis to produce lactic acid, causing the pH value of the meat to drop [21]. When the pH value decreases to the isoelectric point of the protein, the proteins attract each other, and the static charge is zero, which reduces their attraction to water. Consequently, protein solubility decreases, and PL increases. At this stage, moisture loss is most serious and the WHC is the worst [22]. Additionally, an excessively low pH can lead to denaturation and cross-linking of proteins. Furthermore, a reduction in the distance between myofibrils, shortening of sarcomeres, and an increase in the interfibrillar space also contribute to moisture loss [23]. As the aging process continued, the pH value increased slightly from 3–7 d, but the difference was not significant, and it increased to 5.62 at 7 d.

#### 3.1.2. Changes in Drip Loss

The changes in DL during postmortem aging were shown in Figure 1c, which showed a trend of initially increasing and then decreasing. The DL of yak meat increased significantly during 0.5–3 d after slaughter (*p* < 0.05), reaching a maximum value of 3.19% at 3 d, followed by a significant decrease to 2.37% at 7 d (*p* < 0.05). Severe DL during meat storage, transportation, and processing leads to serious quality loss and quality deterioration, resulting in huge economic losses for meat processing enterprises [24,25]. DL is influenced by various factors, including different processing methods of muscle tissues and biochemical changes, etc. Current studies mostly focus on changes in water channels caused by structural changes, protein modification and denaturation of muscle tissue, which ultimately lead to DL [26].

#### 3.1.3. Changes in Cooking Loss

As shown in Figure 1d, during the entire aging process, the CL exhibited a trend of first increasing and then decreasing. The CL increased significantly with the increase in aging time from 0–3 d after slaughter (*p* < 0.05), reaching a maximum value of 42.82% at 3 d, which was 12.36% higher than that at 0 d. After 3 d, it gradually decreased, dropping to 38.14% at 7 d. CL of beef is also one of the important indexes to measure muscle WHC. There is a phenomenon in which the CL first increases during the entire aging process, reaches the peak at 3 d, and then decreases; this may be attributed to the denaturation of muscle protein after slaughter, the gradual degradation of the muscle skeletal proteins, the destruction of the muscle structure, and the consequent reduction in WHC. Meanwhile, during the early postmortem aging period, the increase in CL may also be attributed to the shrinkage of the myofibrillar network structure induced by a decrease in pH value. Additionally, under cooking conditions, thermal denaturation of muscle proteins causes the contraction of myofibril, reducing the space available for immobilized water, consequently, some of the immobilized water is converted into free water and lost [27]. During thermal processing, WHC is primarily related to the extent of thermal denaturation of myofibrillar proteins. Initially, myofibrils are not completely denatured, the pressure and tension generated by the muscle fibers are small, and the water overflow is small. With the increase in cooking temperature, myofibrils become fully denatured, generating greater pressure and tension, and the water overflow is constant, leading to the significant increase in CL [28].

### 3.2. Summary of Protein Identification Results

Proteomics analysis was performed on yak meat during postmortem aging at 0, 3, and 5 d using TMT-labeled quantitative proteomics. The results were presented in Table 3. LC-MS/MS mass spectrometry identified a total of 6439 peptides and 1239 proteins.

### 3.3. Differential Expression Protein Screening

**Figure 2:**
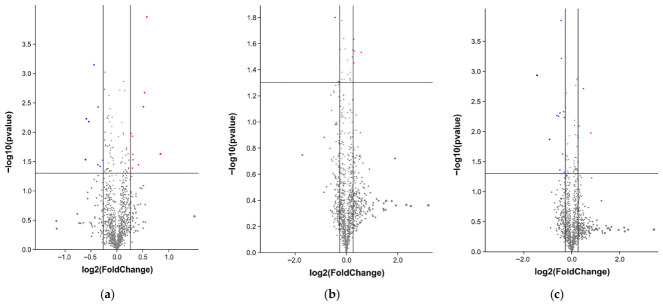
Differential protein abundance distribution in yak meat: (**a**) 3 d vs. 0 d; (**b**) 5 d vs. 0 d; (**c**) 5 d vs. 3 d. Red protein spots represent significantly up-regulated proteins; blue protein spots represent significantly down-regulated proteins; gray protein spots represent proteins that did not change significantly.

The differential proteins were screened based on the criteria of expression fold change (up-regulated more than 1.2 times or down-regulated less than 0.833 times) and *p* < 0.05. The distribution of differential protein expression fold in each group were shown in Figure 2.

This study included three comparison groups: 3 d vs. 0 d, 5 d vs. 0 d, and 5 d vs. 3 d. The numbers of differentially expressed proteins obtained after comparison were shown in Table 4. In group (a), 18 differential proteins were obtained, including 10 up-regulated proteins and 8 down-regulated proteins. In group (b), 6 differential proteins were obtained, including 4 up-regulated proteins and 2 down-regulated proteins. In group (c), 17 differential proteins were obtained, including 5 up-regulated proteins and 12 down-regulated proteins. A total of 41 differentially expressed proteins were identified in this study. Based on their functional characteristics, these proteins were mainly classified into metabolic enzymes, structural proteins, and stress proteins. Metabolic enzymes play an important roles in biochemical reactions such as the tricarboxylic acid (TCA) cycle and glycolysis. Structural proteins, including myosin and actin, are a class of proteins that constitute the intercellular matrix and are closely associated with biological organisms; they are the basis of muscle contraction, maintenance of cell morphology and energy metabolism after slaughter in animals [29]. Stress proteins are a large class of proteins induced by biological organisms to protect cells from damage, and they serve as important regulatory proteins that prevent the degradation of cytoskeletal proteins and maintain the structural stability of myofibrils [30]. The three types of differential proteins were closely related to meat quality such as WHC, tenderness, and meat color.

### 3.4. Clustering Analysis of Differential Expression Proteins

**Figure 3:**
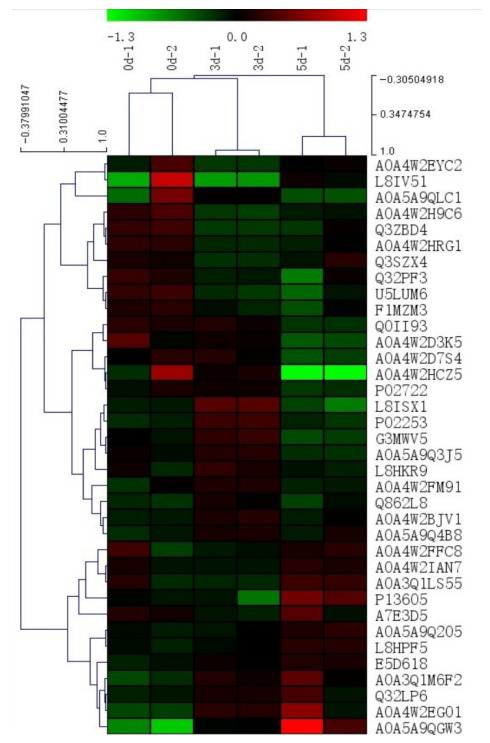
Cluster analysis diagram of differential protein in yak meat. Each row in the figure represents a protein. Red represents relatively high protein abundance, green represents relatively low protein abundance.

The results of hierarchical clustering were presented as a tree-shaped heat map, and the changes in the abundance of differential proteins during the postmortem aging of yak meat were analyzed by hierarchical clustering method. Hierarchical clustering algorithm was used to perform a cluster analysis of the differentially expressed proteins among the three groups. As shown in Figure 3, at lower cluster distances, the two biological replicates samples within each group were first grouped together; this is because the biological replicate has the same aging time point, and the protein expression and function were more similar. With the increase in clustering distance, meat samples from 0 d and 3 d were grouped together and separated from the meat samples at 5 d, indicating a significant change between the early and late stages of postmortem aging in yak meat. With a further increase in cluster distance, all samples were grouped together, indicating that all three samples originated from yak meat and belonged to the same category [31]. Overall analysis revealed that the abundance of differentially expressed proteins in yak meat changed significantly during postmortem aging at 0, 3, and 5 d.

### 3.5. Correlation Analysis of WHC and the Abundance of Differential Proteins

Correlation analysis was conducted between WHC indicators of yak meat and the expression levels of differential proteins. Proteins with significant correlation were identified as key differential proteins affecting the WHC of yak meat. As shown in Table 5, a total of 14 key proteins exhibited significant correlations with the WHC indicators of yak meat. They were classified into 4 groups based on their protein functions: 5 muscle structural proteins (PCNP, SMPX, ITIH1, TTN, STAT3); 4 metabolic enzyme proteins (CA3, PAFAH1B2, CPS1, CAST); 2 stress proteins (HSPB7, GSTO1); 3 other proteins (TMEM38A, CCDC180, HN1).

The most fundamental reason for the change in muscle WHC is the change in muscle tissue structure. The cross-linking, denaturation and degradation of structural proteins play an important role in improving muscle WHC, scholars further pointed out that the degradation of myofibrillar structural proteins in muscle is the main reason for the improvement of meat WHC [32]. Vitale et al. found that the muscle structure becomes less compact during the postmortem aging due to the degradation of myofibrillar and cytoskeletal proteins [33]. The results of this study showed that five types of muscle structural proteins underwent degradation or denaturation during postmortem aging, with changes in their expression levels that were significantly correlated with WHC. PCNP may be involved in the regulation of muscle cell growth cycle [34]. SMPX plays a role in regulating the myofiber network during muscle cell growth, adaptation, and repair, and is responsible for maintaining the structural integrity of myofibers, enabling muscle cells to coordinate their structural and functional states. The expression level of SMPX shows an extremely significant correlation with WHC indicators, so it affects muscle WHC based on its effect on myofiber structure [35]. ITIH1 can serve as a carrier for hyaluronic acid in serum or a binding protein for HA to other matrix proteins, including the regulation of localization, synthesis and degradation of hyaluronic acid on the cell surface of muscle tissue, which are essential for cell biological processes. TTN acts as a molecular template in the assembly of thick and thin filaments, enabling precise regulation of these myofilaments and maintaining the integrity and stability of myofibrils [36]. Structural proteins, including TTN, SMPX, and myofibrillar scaffold proteins, maintain the integrity and arrangement of myofibrils. When these proteins are intact and tightly packed, the myofibrillar space is narrow, forcing water out of muscle cells and leading to high drip loss and cooking loss [37]. During postmortem aging, limited but controlled degradation of structural proteins loosens the myofibrillar network, increases inter-filament spacing, and creates more physical space to immobilize and retain water, thereby improving WHC.

The results of this study showed that the protein expression levels of four metabolic enzymes changed during postmortem aging, which were significantly correlated with WHC. Metabolic enzymes influence glycolysis, ATP consumption, and pH decline rate, indirectly regulating protein solubility and water distribution. CA3 and CPS1 play a critical role in energy metabolism, and the more important pathway of energy metabolism is the glycolysis pathway. CA3 and CPS1 may play an important role in glycolysis, and the rate of glycolysis has a positive effect during postmortem aging. The decrease in pH is the result of the production and accumulation of lactate in the muscle, which facilitates transverse contraction of myofibrils, leading to the extension of sarcomeres and consequently contributing to higher WHC [38]. CAST involved in the biological process was proteolysis regulation (GO:0045861) in this study. It plays a critical role during postmortem aging of meat and is considered to be associated with the degradation of muscle proteins. CAST inhibits the autolysis of calpain and affect the degradation of muscle proteins including desmin, titin and troponin-T by calpain, and further affects muscle WHC [39]. In this study, the expression level of CAST at 3 d was lower than 0 d, indicating that the activity of calpain in yak meat was inhibited by CAST, the degradation of myofibrillar protein was blocked, and the WHC reduced. Meanwhile, the expression level of CAST showed an extremely significant negative correlation with the WHC index, and the correlation coefficients were −0.991 and −0.992.

The results of this study showed that the protein expression of two stress proteins changed during postmortem aging, which were significantly correlated with WHC. Stress proteins such as HSPB1 and GSTO1 maintain protein conformation and reduce oxidative damage, helping stabilize the protein matrix and preserve water-binding sites [40]. Together, these proteins coordinate to maintain cellular integrity and reduce water loss. HSPB1 is a heat shock protein with molecular chaperone function that plays a role in actin organization and HSPB1 may influence muscle WHC by regulating apoptosis [41]. Tao et al. found that a high abundance of HSPB1 contributes to the formation of high WHC [42]. Song et al. found that the low expression levels of HSPB1 reduce the stability of actin filaments, and lead to decreased WHC [43]. In this study, the expression level of HSPB1 at 5 d was higher than 3 d, which well verified the above statement, indicating that the WHC of yak meat at 3 d was lower than at 5 d. GSTO1 plays an important role in cytotoxicity and antioxidant mechanisms. In the GO analysis of this study, the biological processes it participated in were primarily cellular modification and catabolic processes. Suman et al. found that overexpression of GSTO1 in postmortem muscle would accelerate ATP depletion and pH decline, thereby reducing muscle WHC [44].

### 3.6. Bioinformatics Analysis of Key Proteins

#### 3.6.1. GO Analysis

**Figure 4:**
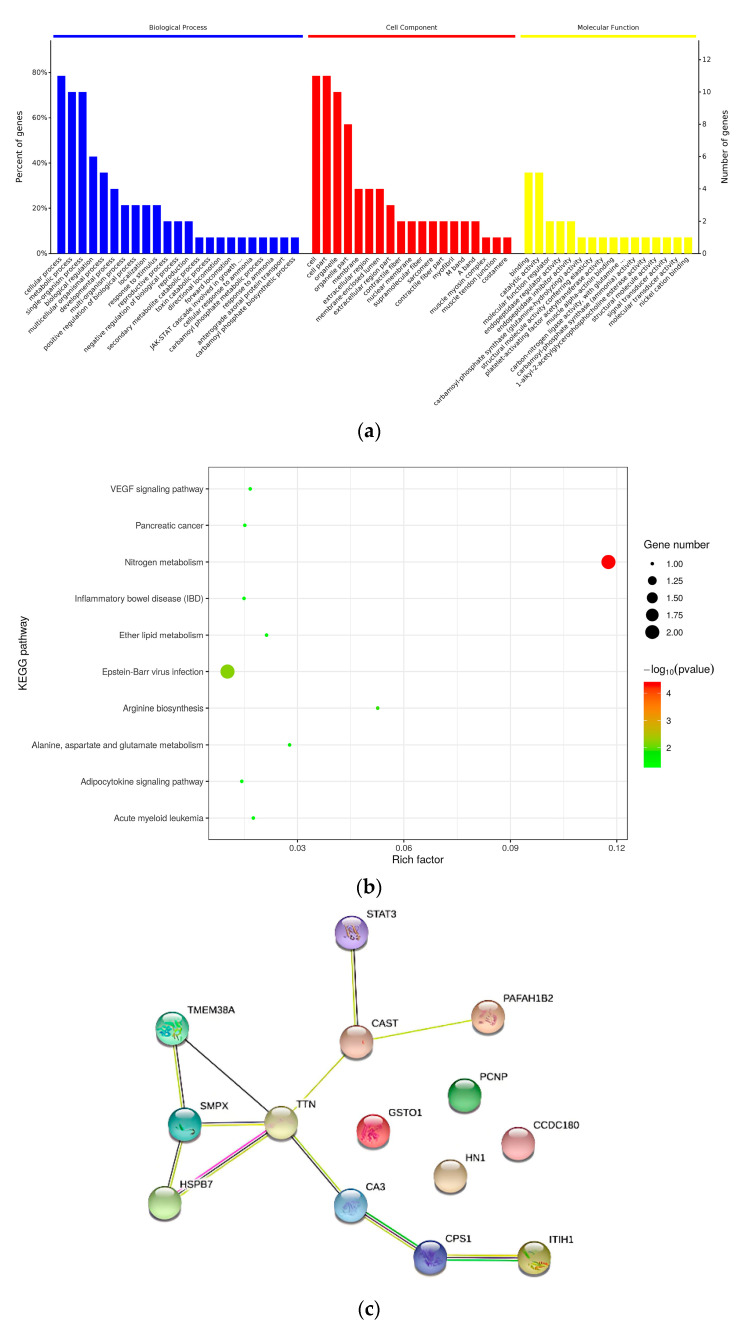
Bioinformatics analysis of key proteins in yak meat. (**a**) GO analysis. The abscissa in the graph represents the GO functional classification, including BP, CC and MF. (**b**) KEGG analysis. The abscissa represents the enriched factors for each KEGG pathway, the color of the bubbles indicates the significance of the KEGG pathway, the size of the bubbles indicates the number of differentially expressed proteins involved in the KEGG pathway. (**c**) Protein interaction networks of key proteins. The number of proteins that directly interact with a protein is called the connectivity degree of that protein.

As shown in Figure 4a, the key proteins involved in biological processes mainly included carbamoyl phosphate biosynthesis, carbamoyl phosphate metabolism process, striated muscle contraction, arginine biosynthesis process, pyrimidine nuclease biosynthesis process, myosin filament assembly, striated muscle myosin thick filament assembly, cellular modified amino acid metabolism process, growth hormone receptor signaling pathway, glycogenolysis process, muscle contraction, negative regulation of endopeptidase activity, lipid catabolic process, glutamine metabolism processes, muscular system processes, single-organism catabolic processes, negative regulation of proteolysis, and cellular catabolic processes.

Cellular components in which the key proteins were located mainly include M-line, A band, sarcomere, nuclear envelope, sarcoplasmic reticulum membrane, nucleus, mitochondrial nucleoid, sarcoplasm, intracellular non-membrane-bounded organelle, and myosin complex.

Molecular functions of the key proteins mainly included glutamine hydrolase activity, 1-alkyl-2-acetylglycerophosphocholine esterase activity, structural molecule activity conferring elasticity, peptide chain endonuclease inhibitor activity, muscle α-actinin binding, peptidase regulatory activity, calcium-activated potassium channel activity; muscle structural components, protein binding including protein folding, α-actinin binding, calcium-activated cation channel activity, and modified amino acid binding.

#### 3.6.2. KEGG Analysis

KEGG pathway analysis was conducted on the key proteins affecting the WHC of yak meat, elucidating the metabolic pathways in which these key proteins were involved. As shown in Figure 4b, the key proteins were mainly involved in 12 metabolic pathways. Among them, nitrogen metabolism (bta00910) involved 2 proteins; EB virus infection (bta05169) involved 2 proteins; arginine biosynthesis (bta00220) involved 1 protein; alanine, aspartate and glutamate metabolism (bta00250) involved 1 protein; ether lipid metabolism (bta00565) involved 1 protein; acute myeloid leukemia (bta05221) involved 1 protein; vascular endothelial growth factor signaling pathway (bta04370) involved 1 protein; pancreatic cancer (bta05212) involved 1 protein; inflammatory bowel disease (bta05321) involved 1 protein; adipocytokine signaling pathway (bta04920) involved 1 protein; amino acid biosynthesis (bta01230) involved 1 protein; and prolactin signaling pathway (bta04917) involved 1 protein.

In summary, the 14 key proteins affecting the WHC of yak meat were primarily involved in 4 categories of metabolic pathways: energy metabolism, lipid metabolism, amino acid metabolism, and amino acid biosynthesis.

#### 3.6.3. Protein Interaction Networks

The interaction network of key proteins in yak meat is shown in Figure 4c. The connectivity degree of TTN was 5, showing strong interactions with the structural proteins SMPX and HSPB1, the metabolic enzymes CAST, CA3, and TMEM38A; the connectivity degree of SMPX was 3, showing strong interactions with the structural protein HSPB1, TMEM38A, and TTN; the connectivity degree of CAST was 3, showing strong interactions with the structural protein STAT3, the metabolic enzyme PAFAH1B2, and TTN. Highly aggregated and highly connected proteins may be key points that affect the whole system or metabolic pathways [45]. Combined with the analysis in Figure 4a, TTN is involved in the biological processes of myosin filament assembly and myofibril assembly, and the molecular functions involved include structural molecular elasticity and actin binding [46]. SMPX is associated with cellular components including the A band, M line, sarcomere, contractile fiber, etc. [47]. The biological processes involved in CAST include the negative regulation of proteolysis, and the molecular functions involved include the activity of peptide chain endonuclease inhibitors [48]. In summary, the biological functions of the aforementioned proteins involve the changes in muscle fiber structure and muscle structural proteins, and such changes were closely associated with alterations of muscle WHC.

### 3.7. Parallel Reaction Monitoring (PRM) Verification

The seven key proteins of WHC (SMPX, TTN, STAT3, CA3, CAST, GSTO1, JPT1) and five differential proteins (PSMA7, HIST1H1D, MYBPH, HIST1H1E, COL1A1) that met the PRM verification conditions were verified, and the results were shown in Table 6. For the 12 proteins in yak meat that met the PRM analysis criteria, the fold changes in up-regulation and down-regulation in protein expression levels across each comparison group were consistent with the TMT analysis results, and the results of PRM verification were completely consistent with the results of TMT analysis, demonstrating the reliability of the TMT-based quantitative proteomics analysis.

## 4. Conclusions

In this study, the key proteins affecting the WHC of yak meat and the associated metabolic pathways were identified. Through correlation analysis, 14 target proteins significantly correlated with the WHC of yak meat were screened from 41 differential proteins, which were divided into three categories according to their functions: metabolic enzymes, structural proteins and stress proteins. Bioinformatics analysis of the target proteins indicated that energy metabolism, lipid metabolism, amino acid metabolism, and amino acid biosynthesis were main metabolic pathways affecting the WHC of yak meat. Key protein interaction analysis combined with bioinformatics analysis showed that TTN, CAST, SMPX may serve as indicator proteins for the WHC of yak meat during postmortem aging. PRM validation of seven target proteins and five differential proteins demonstrated that TMT-based quantitative proteomic analysis was highly reliable for analyzing muscle WHC. Future studies should conduct in-depth investigations into how interactions among key proteins influence muscle WHC and clarify how key proteins function in metabolic pathways.

## Figures and Tables

**Table 1:** The marker information of the peptide.

TMT Marking	126	127	128	129	130	131
Sample Name	0 d-1	0 d-2	3 d-1	3 d-2	5 d-1	5 d-2

**Table 2:** The parameters of protein identification and quantitative analysis.

Item	Value
Type of search	MS/MS Ion search
Enzyme	Trypsin
Mass Values	Monoisotopic
Max Missed Cleavages	2
Fixed modifications	Carbamidomethyl (C), TMT 10plex (K)
Variable modifications	Oxidation (M), TMT 10plex(Y)
Peptide Mass Tolerance	±20 ppm
Fragment Mass Tolerance	0.1 Da
Protein Mass	Unrestricted
Database	uniprot_Bos_152013_20191114
Database pattern	Decoy
FDR	≤0.01
Protein Quantification	The protein ratios are calculated as the median of only the unique peptides of the protein
Experimental Bias	Normalizes all peptide ratios by the median protein ratio; the median protein ratio should be 1 after normalization

**Table 3:** Protein identification results.

Database	Total Spectra	Spectra (PSM)	Peptides	Protein Groups
Bos	140,242	24,246	6439	1239

Database: database species names. Total spectra: total number of secondary mass spectrograms. Spectra: the number of spectra to match the peptides. Peptides: the total number of peptides identified. Protein groups: the total number of proteins identified.

**Table 4:** Results statistics of protein quantitative and variance analysis.

Comparisons	Up	Down	All
3 d vs. 0 d	10	8	18
5 d vs. 0 d	4	2	6
5 d vs. 3 d	5	12	17

Comparisons: different comparison groups; Up: up-regulated proteins; Down: down-regulated proteins; All: all differentially expressed proteins.

**Table 5:** Correlation analysis of WHC and differential protein abundance in yak meat.

Number of Proteins	Protein Names	Correlation Coefficient		
		CL	DL	PL
Structural Protein				
Q32PF3	Proteolytic signal-containing nuclear protein (PCNP)	−0.967 **	0.767	−0.965 **
Q3ZBD4	Small muscular protein (SMPX)	−0.983 **	−0.968 **	−0.984 **
A0A4W2BJV1	Inter-alpha-trypsin inhibitor heavy chain H1 (ITIH1)	0.730	0.951 **	0.736
A0A3Q1M6F2	Titin isoform X3 (TTN)	0.937 *	0.701	0.934 *
Q32LP6	Signal transducer and activator of transcription (STAT3)	0.991 **	0.839	0.990 **
Metabolic Enzymes				
Q3SZX4	Carbonic anhydrase 3 (CA3)	0.682	−0.928 *	0.688
Q862L8	Platelet-activating factor acetylhydrolase, β subunit (PAFAH1B2)	0.641	0.906 *	0.647
A0A4W2EG01	Carbamoyl-phosphate synthase 1 (CPS1)	0.942 *	0.710	0.939 *
U5LUM6	Calpastatin (CAST)	−0.992 **	0.846	−0.991 **
Stress Protein				
A0A4W2EYC2	Heat shock protein beta-1 (HSPB1)	0.771	−0.968 **	0.776
A0A4W2HRG1	Glutathione s-transferase omega 1 (GSTO1)	−0.982 **	−0.969 **	−0.984 **
Other Proteins				
A0A5A9Q4B8	Trimeric intracellular cation channel type A (TMEM38A)	0.909 *	1.000 **	0.912 *
F1MZM3	Coiled-coil domain containing 180 (CCDC180)	−0.972 **	0.781	−0.970 **
A0A4W2H9C6	Jupiter microtubule associated homolog 1 (HN1)	−0.983 **	−0.968 **	−0.984 **

Note: ** means the differences between WHC indicators and the expression levels of differential proteins (*p* < 0.01); * means the differences between WHC indicators and the expression levels of differential proteins (*p* < 0.05).

**Table 6:** PRM verification of differentially expressed proteins.

Serial Number	Name	TMT		PRM	
		Ratio 3 d/0 d	Ratio 5 d/3 d	Ratio 3 d/0 d	Ratio 5 d/3 d
Q3ZBD4	SMPX	0.66 ↓	-	0.34 ↓	-
A0A3Q1M6F2	TTN	1.34 ↑	-	1.54 ↑	-
Q32LP6	STAT3	1.21 ↑	-	1.26 ↑	-
Q3SZX4	CA3	0.78 ↓	-	0.23 ↓	-
U5LUM6	CAST	0.69 ↓	-	0.69 ↓	-
A0A4W2HRG1	GSTO1	0.74 ↓	-	0.76 ↓	-
A0A4W2H9C6	JPT1	0.66 ↓	-	0.75 ↓	-
A7E3D5	PSMA7	0.83 ↓	-	0.77 ↓	-
P02253	HIST1H1D	1.43 ↑	0.68 ↓	1.47 ↑	0.51 ↓
L8ISX1	MYBPH	1.49 ↑	0.53 ↓	1.25 ↑	0.19 ↓
G3MWV5	HIST1H1E	1.24 ↑	0.65 ↓	1.64 ↑	0.72 ↓
L8IV51	COL1A1	-	1.74 ↑	-	1.72 ↑

Note: The arrows indicate the upregulation and downregulation of protein expression levels by a certain multiple.

## Data Availability

The data generated from the study are clearly presented and discussed in the manuscript, further inquiries can be directed to the corresponding author.
